# Selenium alters the gene content but not the taxonomic composition of the soil microbiome

**DOI:** 10.1186/s40793-024-00641-x

**Published:** 2024-11-18

**Authors:** Alison E. Bennett, Scott Kelsey, Casey Saup, Mike Wilkins, Antonino Malacrinò

**Affiliations:** 1 Department of Evolution, Ecology, and Organismal Biology, The Ohio State University, Columbus, OH USA; 2 School of Earth Sciences, The Ohio State University, Columbus, OH USA; 3 Department of Soil and Crop Sciences, Colorado State University, Fort Collins, CO USA; 4 Department of Agriculture, Università degli Studi Mediterranea di Reggio Calabria, Reggio Calabria, Italy; 5 Department of Biological Sciences, Clemson University, Clemson, SC USA

**Keywords:** Shotgun metagenomics, Amplicon sequencing, Heavy metal

## Abstract

**Background:**

Microbiomes, essential to ecosystem processes, face strong selective forces that can drive rapid evolutionary adaptation. However, our understanding of evolutionary processes within natural systems remains limited. We investigated evolution in response to naturally occurring selenium in soils of different geological parental materials on the Western Slope of Colorado. Our study focused on examining changes in gene frequencies within microbial communities in response to selenium exposure.

**Results:**

Despite expectations of taxonomic composition shifts and increased gene content changes at high-selenium sites, we found no significant alterations in microbial diversity or community composition. Surprisingly, we observed a significant increase in differentially abundant genes within high-selenium sites.

**Conclusions:**

These findings are suggestive that selection within microbiomes primarily drives the accumulation of genes among existing microbial taxa, rather than microbial species turnover, in response to strong stressors like selenium. Our study highlights an unusual system that allows us to examine evolution in response to the same stressor annually in a non-model system, contributing to understanding microbiome evolution beyond model systems.

**Supplementary Information:**

The online version contains supplementary material available at 10.1186/s40793-024-00641-x.

## Background

Natural selection and ecological community assembly are forces that act on all large and small organisms. However, microbial communities are particularly exciting systems for exploring community assembly and evolutionary adaptation because they are easily manipulated and experience strong selective forces. Here we define evolution as changes in the frequency of alleles and genes. Significant research has demonstrated that strong stressors (e.g., antibiotics) drive both changes in community composition and evolutionary adaptation [e.g., 1–5]. Strong forces are also expected to drive adaptation in multiple different taxa simultaneously within a given microbiome. Although evolutionary adaptation has been tracked for specific microbial groups within gut systems, fewer studies have addressed these questions in non-gut natural systems [6, but see 7].

Microbiomes, defined here as heterogeneous communities of functionally diverse microbes of both prokaryotic and eukaryotic origin occupying the same physical space, are essential to ecosystem processes. Microbiomes assemble through a continual complex interplay between microbes and their environment that leads to the success of some species (and genotypes within species) and the loss of others. To date, little is known about the evolutionary processes that underpin changes in the interactions within and the composition of microbiomes [8–10]. Here, we examine both microbiome composition and evolution within microbes within microbiomes, defined as changes in gene frequencies within a microbial community in response to an external force.

Based on previous research, we expect strong stresses to drive changes in microbiome community composition. Most studies in most systems (e.g., guts, plants, water [11–14]) report changes in the community composition of the microbiome between stress treatments, and these changes are reported in laboratory manipulations as well as natural systems. Functional redundancy may occur, allowing functions to be maintained despite shifts in composition. Shifts in microbial community composition are likely driven by the elimination of species that cannot tolerate the stress, and based on macrosystems and previous work in microbiomes, we expect shifts in community composition to occur before evolutionary adaptation.

We expect the ability of microbes within microbiomes to adapt rapidly to stress to alter microbiome function. For example, in human microbiomes, rapid adaptation has allowed the digestion of new foods and diets [15]. In non-host systems, adaptation within microbiomes should allow the persistence of microbes in the face of stress as well as the development of unique functions to promote stress tolerance. Microbiome evolution and adaptation may be most important in the face of transient stresses such as drought or exposure to heavy metals.

Changes in gene variant frequencies within a microbial community have been observed in the gut microbiomes of humans and other species [e.g., 15]. In the human gut microbiomes, the mutation rate can reach as high as 10^9^-10^12^ new single nucleotide polymorphisms (SNPs) recorded per day [16]. Fewer examples exist for non-host systems, but there is evidence of evolution. For example, a nine-year metagenomic study of a freshwater lake showed evidence of selection and hitchhiking of genes in genomic sweeps [17]. As mentioned above, selective sweeps have also been observed in soil microbiomes in just two months [18]. Chase et al. [19] tracked genomic mutations of a common member of a soil decomposer community transplanted to sites across a climatic gradient. They found a number of mutations associated with transplantation. These are all clear examples of rapid evolution within a microbiome, although most of the studies in natural systems have documented selection in response to a suite of selective pressures (e.g., changes in climatic variables). To our knowledge, no study of a natural system has examined evolution within a microbiome in response to a single known selective pressure.

To address this gap, we examined evolution within a microbiome in response to exposure to naturally occurring selenium (Se), a heavy metal, in soils of different parental materials on the Western Slope of Colorado. Se can be highly toxic for microbes, plants, and mammals such as cattle [20, 21], acting as a strong selective force for a wide range of organisms in natural systems. In most of our study sites, Se appears in early spring with the introduction of water via rain and snowmelt and has disappeared from soil by May. Thus, Se is an excellent model system as it creates a strong temporal selective pressure on the microbes in the soil that allows observation of evolutionary responses annually. Other heavy metals usually persist in the soil throughout the year, and thus we expect evolutionary responses to other heavy metals to be historical changes and not indicative of recent selective events. On the Western Slope, multiple geological profiles are exposed, creating a patchwork of soils of different parental materials as well as a patchwork of Se [22]. This provides an excellent opportunity to explore the influence of Se in pairs of soils from the same geological profile. To assess evolution within a microbiome in response to Se, we conducted metagenomic analyses to explore the changes in gene frequency driven by Se and underlying parent material. We hypothesized that we would see changes in taxonomic composition as bacteria without the ability to tolerate Se were eliminated. We also hypothesized that we would detect greater changes in gene content at sites with Se and that genes with recorded changes would be related to Se metabolism or tolerance.

## Methods

### Sampling

**Fig. 1:**
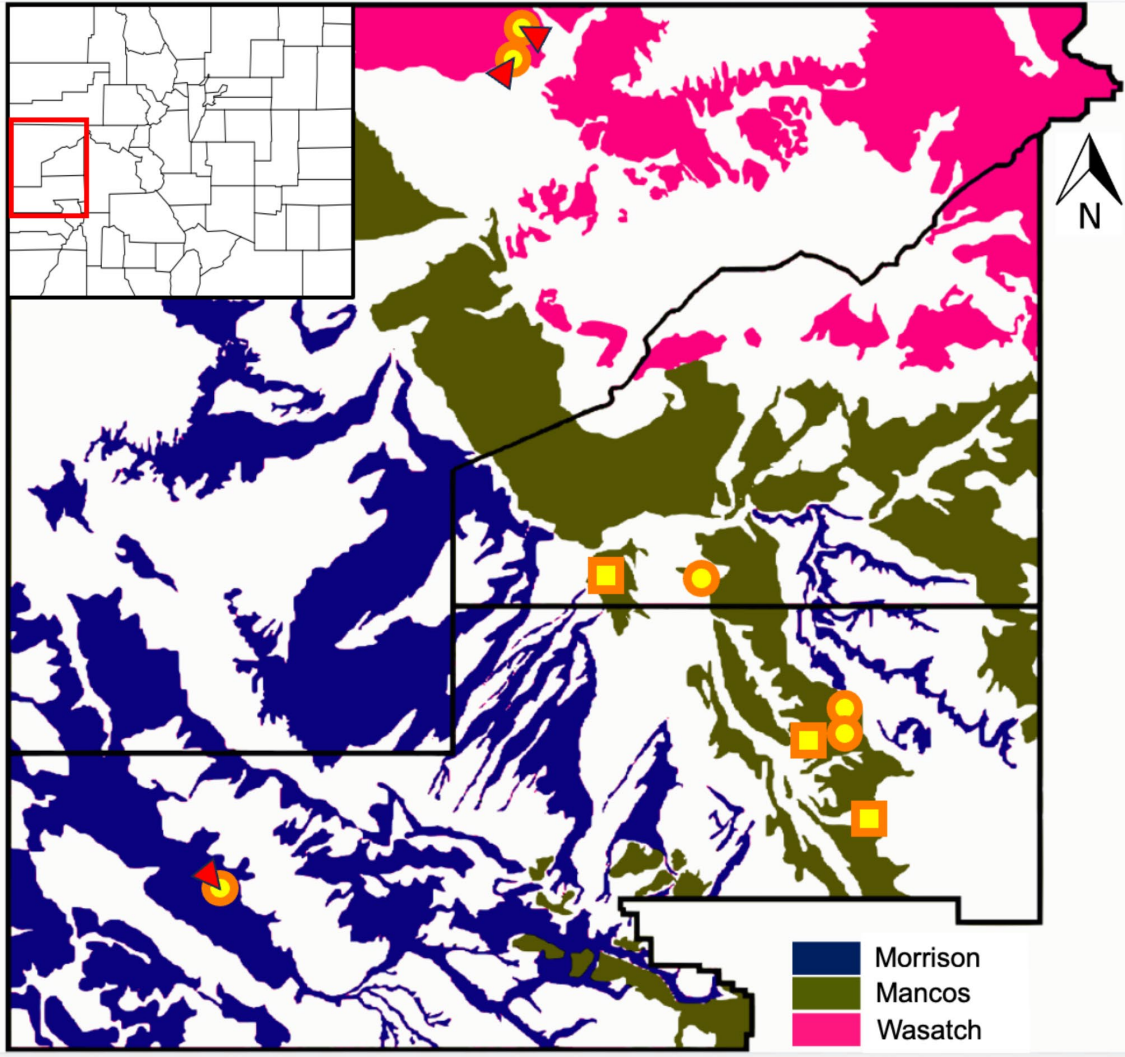
Map of paired high/low selenium sampling sites in Western Colorado, USA. Colors correspond to the underlying geological parent material responsible for the formation of high-Se soils. Shapes indicate paired high- (circle) and low- (square) selenium soils, while points marked with red triangles indicate areas where samples from both high- and low-selenium soils were collected. Counties are Mesa, Delta, and Montrose Counties from north to south. The inset map on the top left highlights the locations of counties in Colorado. See Tab. S1 for further details

Soils with high concentrations of Se naturally occur throughout the southwestern US, but the greatest concentration of Se sites derived from multiple geological formations are located on the Western Slope, CO, where we focused our sampling efforts. We sampled three paired 1 m × 1 m plots of high and low Se spread across three different geological formations (Mancos, Wasatch, and Morrison) in CO (Fig. 1, Tab. S1) for a total of 19 plots (high *n* = 9, low *n* = 10). Se enters these sites in water in spring (March-April) and disappears from the soil at most of the sites by mid-May (personal observations). This is similar to other systems which have shown that water introduces Se into soil systems, but organic matter is necessary to retain Se within soils [23, 24]. These three soil formations give rise to different soil types [25] —all of which are extremely low in organic matter. Morrison soils are derived from sedimentary rocks from the Upper Jurassic period and contain mudstone, sandstone, siltstone, and limestone, and this combination of softer rock sources produces a sandier gray, green, or red soil with a high fine clay content [26, 27]. Mancos soils are derived from sedimentary rocks from the Upper Cretaceous period and contain predominantly mudstone, which produces primarily gray silty soil [28]. Wasatch soils were deposited in a delta, as evidenced by the great variety of soil colors (bright red, purple, yellow and gray) in clear stripes along hillsides. These soils have a similar composition to Morrison soils but vary more in color [29]. These sites all occur in a desert or desert-like habitat with minimal vegetation.

Across a one week interval in early May 2019, at each site, we collected and sieved (0.5 mm mesh) soil from a 1 m^2^ area surrounding a piece of rebar permanently placed at the center of each plot. From this sample, we preserved ~ 100 g of homogenized bulk soil by immediately flash-freezing it in an ethanol/dry ice cooling bath (-72 °C) and storing it on dry ice until final storage at -80 °C. The bedrock at these sites can be very shallow, so where possible, soil was collected from the top 15 cm of soil.

### Selenium quantification

Selenium is transient in soil as it primarily exists as a highly soluble ion and is quickly leached by water. Even at high-Se sites, we can rarely detect Se in soil during our sampling time (May). However, Se is persistent in plant material; thus, we analyzed plant material to determine high and low levels of Se at each site. There was no common plant present at all the sites, and the amount of Se within plant tissues varied by plant species; therefore, we assessed the amount of Se in the samples from three different plant species per site. We acknowledge that this is an imperfect measurement, and we thus do not relate measured quantities of Se in plant tissue to soil concentrations or include them in further analyses. Instead, we use Se in plant tissue to confirm that our sites are relatively high (above a threshold) or low in Se. Thus, the plant samples were dried at 60 °C, ground, and 0.15 g of plant material was digested in 2 mL of analytical grade HNO_3_ for 30 min at 90 °C. Then, 1 mL of 30% H_2_O_2_ was added, and the samples were digested for an additional 30 min at 90 °C. After cooling, we diluted the samples to 10% v/v acid and heated them for an additional 90 min at 90 °C. Samples were then filtered, and 4.85 mL of the filtered sample was combined with 0.15 mL of ethanol and the internal standard Tellurium. These samples were then run on a Perkin-Elmer Optima 4300DV Inductively Coupled Plasma Optical Emission Spectrometer (Shelton, CT, USA) with the analytical line set at 196.026 nm, and compared to a calibration curve to identify the Se concentration of each sample.

### DNA extraction, library preparation, and sequencing

DNA was extracted in triplicate from each sample and each aliquot was then pooled back into an individual sample before library preparation (19 samples in total – high Se *n* = 9, low Se *n* = 10). Briefly, ~ 50 mg of each sample was mixed with 0.2 g of zirconia beads and two stainless steel beads (diam. 2.4 mm) and dry milled for 5 min at 30 Hz using a TissueLyzer II (Qiagen). Then, the samples were milled again after adding 500 µL of lysis buffer (10 mM Tris, 100 mM NaCl, 10 mM EDTA, and 0.5% sodium dodecyl sulfate) and milled again for 5 min at 30 Hz. After brief centrifugation, we added 2 µL of metapolyzime (Sigma‒Aldrich) to further enhance microbial lysis. Then, DNA was extracted using phenol: chloroform extraction, purified using Sera-Mag Speedbeads (Cytiva), and checked using a Nanodrop One spectrophotometer (Thermo) for quantity and purity. DNA extractions included non-template controls (in triplicate) where we replaced the soil samples with 300 µL of nuclease-free water.

Libraries for shotgun metagenomics were prepared using the NEBNext Ultra II FS DNA library prep kit for Illumina (New England Biolabs) according to the supplier’s recommendation, checked using a Bioanalyzer HS DNA chip (Agilent), quantified using a Qubit fluorometer (Thermo), pooled at equimolar ratios, and sequenced using a HiSeq 4000 instrument (Illumina, San Diego, CA, USA) using PE150 chemistry.

### Data processing and analysis

The raw data were processed using TrimGalore v0.6.7 (https://github.com/FelixKrueger/TrimGalore) to remove Illumina adaptors and discard low-quality reads.

First, we used this dataset to characterize the taxonomic composition of the soil microbial communities at different sites using Kraken2 v2.1.2 [30]. The Kraken2 output was used to estimate the abundance of each taxon using Bracken v2.7 [31]. The data were then processed using R v4.1.2 [32] and phyloseq v1.38 [33]. All singletons were then discarded. Data was rarefied at even depth (90% of the minimum sample depth) before diversity analysis. The Shannon diversity index was calculated using the package microbiome v1.16 [34], and differences between high- and low-selenium soils were tested by fitting a linear mixed-effects model with the package lme4 [35] using the selenium level (high or low) as a fixed factor and geological formation as a random effect. We also tested the effect of the selenium level (high or low) on the structure of microbial communities using PERMANOVA on a Bray‒Curtis distance matrix between samples (999 permutations). Considering that geological formation (Mancos, Wasatch, and Morrison) explained a wide portion of the variance in taxonomic composition (9.62% in the shotgun metagenomics dataset and 31.92% in the amplicon sequencing dataset; see supplementary materials), we stratified permutations using the variable “geological formation” to account for this component in PERMANOVA analyses. Differences in the structure of the soil microbial communities were visualized using Non-metric Multi-dimensional Scaling (NMDS). The data (non-rarefied) were then normalized using Wrench [36], and we identified taxa with differential abundance between soils with high and low Se using the package MaAsLin2 [37].

Second, we used the clean raw sequencing reads to infer the functional composition (in terms of gene content) of the soil microbial communities at different sites. Reads were merged into contigs using MegaHit v.1.2.9 [38], and functional annotation was performed using Prokka v.1.14.6 [39]. The raw reads were mapped against the Prokka output using Bowtie2 and SAMtools [40, 41] to construct a count matrix of the gene frequency for each sample. As above, the data were normalized using Wrench, and using the package MaAsLin2, we identified the genes with differential abundance between soils with high and low Se. The annotation of genes was further refined using the BacMet database [42, accessed on 11 August 2023] and those reported in the previous study by Wang et al. [43].

## Results

Sites with plants containing less than 3 mg Se/kg were categorized as low-Se, and high-Se sites had plants with Se contents ranging from 10 to 30 mg Se/kg [see also 22]. The high- and low-Se sites were in accordance with our sampling expectations (Fig. 1, Tab. S1). As this imperfect measurement of Se was only to confirm our site classifications we did not incorporate Se concentrations into further analyses.

**Fig. 2:**
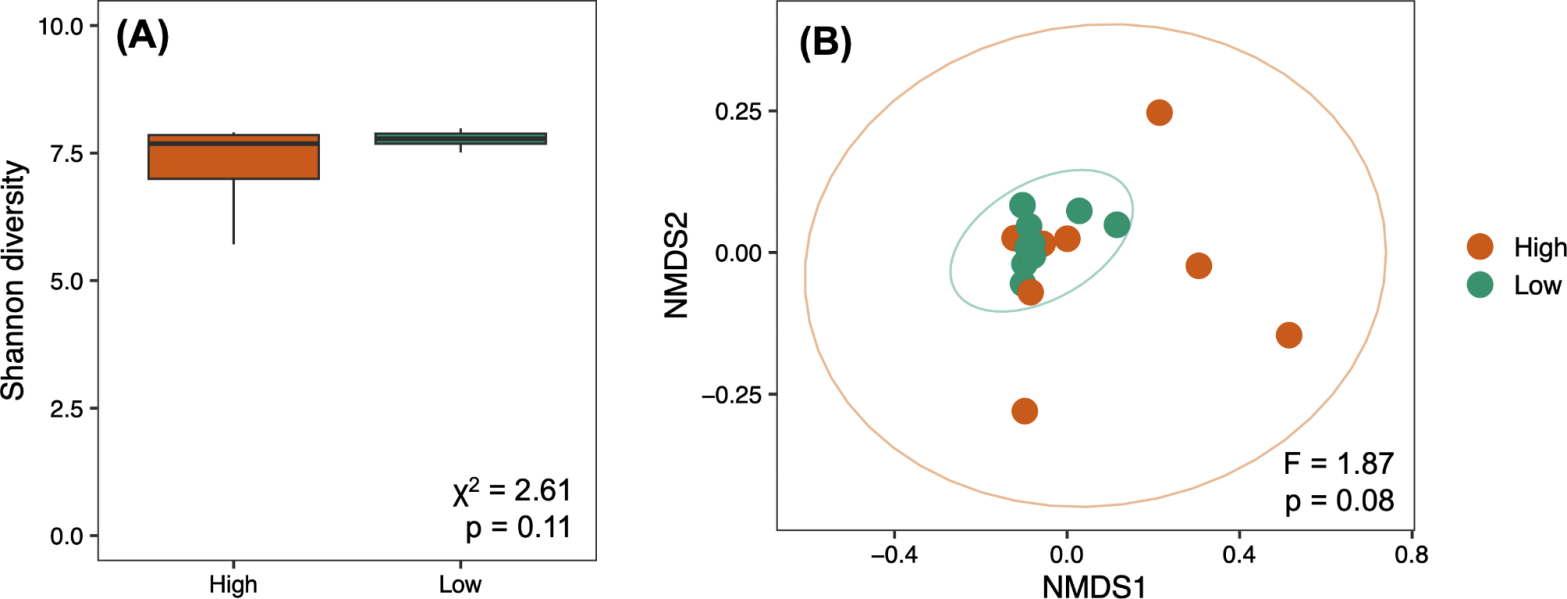
**(A)** Shannon diversity index in soil samples with high (*n* = 9) and low (*n* = 10) Se, with results from a linear mixed-effects model. **(B)**. NMDS (Non-Metric Multi-Dimensional Scaling) of the soil samples with high (*n* = 9) and low (*n* = 10) Se levels, with results from PERMANOVA. The ellipses represent the 95% CIs for each group

**Fig. 3:**
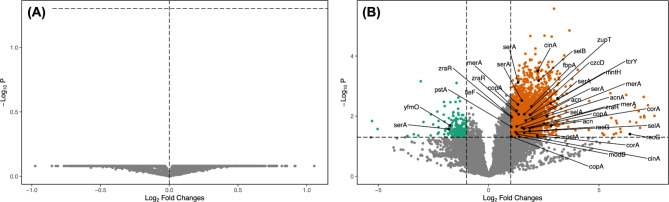
Volcano plots showing **(A)** that no microbial taxa were differentially abundant between sites with high (log2FC > 0) and low (log2FC < 0) Se levels. **(B)** Genes that were more frequent at sites with high (log2FC > 0, orange) or low (log2FC < 0, green) levels of selenium. In panel B, labeled genes are those included in the BacMet database

We generated shotgun metagenomics data from 19 sites (high Se *n* = 9, and low Se *n* = 10), yielding 482,406,146 paired end reads (average 25,389,797 reads per sample). We first focused on testing the influence of selenium on the taxonomic composition of the soil microbial communities. All rarefaction curves flattened out (Fig. S1), and we found that the soil community was mainly dominated by *Pseudomonas*, *Sphingomonas*, *Streptomyces*, and *Cutibacterium* (Fig. S2). Our results showed that the presence of high levels of Se did not influence the diversity (Shannon diversity index, 𝛘^2^ = 2.61, *p* = 0.11; Fig. 2A) or structure (PERMANOVA, F_1, 17_=1.87, *p* = 0.08; Fig. 2B) of the soil microbial communities. Similarly, when testing for differences in individual ASVs between the two soil groups, we did not find any taxa that were significantly more abundant in soils with high or low levels of Se (Fig. 3A). Similar results were obtained using an amplicon sequencing approach from samples collected in 2018 (see Supplementary Results).

We identified a total of 35,232 genes in our dataset including 107 (Table 1) that were annotated as genes coding for selenium resistance in previous studies [43]. We then focused on testing whether high levels of Se influence the gene content of soil microbial communities. Overall, we found 3,133 genes with higher relative abundances in soils with high Se, while 1,440 had higher relative abundances in soils with low Se (Fig. 3B). Among the differentially abundant genes, 36 genes matched genes associated with selenium resistance (Fig. 3B; Table 2), 34 displayed higher relative abundances in soils with high selenium (Fig. 3B).

**Table 1:** List of genes from the BacMet database [42] and Wang et al. [43] that have been reported to contribute to resistance to selenium. For each gene identified in our dataset, we reported the number of gene variants identified within the metagenome and the annotations from Prokka (identified with the symbol ^†^) or Wang et al. (identified with the symbol *)

Gene	Number of gene variants	Annotation
*serA*	27	D-3-phosphoglycerate dehydrogenase^†^
*ruvB*	23	Holliday junction ATP-dependent DNA helicase^†^
*recG*	22	ATP-dependent DNA helicase^†^
*selA*	10	L-seryl-tRNA(Sec) selenium transferase^†^
*serC*	9	Phosphoserine aminotransferase^†^
*selB*	7	Selenocysteine-specific elongation factor^†^
*serB*	6	Phosphoserine phosphatase^†^
*sodB*	2	Superoxide dismutase^†^
*sodA*	1	Superoxide dismutase^†^
*tehB*	-	S-adenosyl-L-methionine-dependent methyltransferase*
*mmtA*	-	Methyltransferase*
*ubiE*	-	Methyltransferase*
*serD*	-	Selenate reductase*
*srdA*	-	Selenate reductase*
*srdB*	-	Selenate reductase*
*srdC*	-	Selenate reductase*

**Table 2:** List of genes from the BacMet database [42] and Wang et al. [43] that were identified as differentially abundant between sites with high and low Se levels in our study. For each gene, we report the number of gene variants identified within the metagenome, the annotation from Prokka, and the metal toward which they confer resistance according to the BacMet database [42] or Wang et al. [43]

Gene	Number of gene variants	Annotation	Metal
*acnA*	3	Aconitate hydratase A	Iron
*cinA*	2	Putative competence-damage inducible protein	Copper
*copA*	3	Copper resistance protein A	Copper
*corA*	2	Magnesium transport protein CorA	Magnesium Cobalt Nickel Manganese
*czcD*	1	Cadmium, cobalt and zinc/H(+)-K(+) antiporter	Cadmium Zinc Cobalt
*fbpA*	1	Fe(3+)-binding periplasmic protein	Iron Gallium
*fieF*	1	Ferrous-iron efflux pump FieF	Nickel Iron Cadmium Cobalt Zinc
*merA*	3	Mercuric reductase	Mercury
*mntH*	1	Divalent metal cation transporter MntH	Manganese Iron Cadmium Cobalt Zinc
*modB*	1	Molybdenum transport system permease protein	Tungsten Molybdenum
*pstA*	2	Phosphate transport system permease protein PstA	Arsenic
*recG*	2	ATP-dependent DNA helicase RecG	Chromium Tellurium **Selenium**
*selA*	2	L-seryl-tRNA(Sec) selenium transferase	**Selenium**
*selB*	1	Selenocysteine-specific elongation factor	**Selenium**
*serA*	5	D-3-phosphoglycerate dehydrogenase	**Selenium**
*tcrY*	1	putative sensor histidine kinase TcrY	Copper
*yfmO*	1	Multidrug efflux protein YfmO	Copper
*zraR*	3	Transcriptional regulatory protein ZraR	Zinc
*zupT*	1	Zinc transporter	Nickel Iron Cadmium Cobalt Zinc Copper

## Discussion

### No changes in community composition

In contrast to our first hypothesis, we found no changes in microbial diversity or community composition across two years (2018 for amplicon sequencing and 2019 for shotgun metagenomics) or across analytical approaches (metabarcoding or metagenomics). In most cases of exposure to strong selective pressures, the literature has reported changes in microbial diversity and composition in multiple systems. In fact, changes in soil microbial diversity and composition in response to Se have also been reported in previous studies [44, 45]. Thus, we were surprised by the lack of changes in composition. However, this is the first study of the impacts of Se on soil microbial diversity to include replicate sites and incorporate geological profile as a predictor.

There are several potential explanations for why our results contrast with those of Rosenfeld et al. [45] and Cochran et al. [44], who reported that Se alters the taxonomic structure of soil microbial communities. Our study focuses on a wider sampling area with different underlying geology, while both Rosenfeld et al. [45] and Cochran et al. [44] collected samples from high- and low-selenium sites with similar underlying geological formations. This sampling approach may have limited their ability to account for differences in microbial communities driven by other factors. In our study, we sampled a wider area that included three different geological formations, and this information allowed us to test whether Se influences the relative abundance of specific microbial taxa, regardless of the effect of the sampling site. In addition, in communities of macroorganisms (e.g., plants and animals), we have frequently observed that selection eliminates taxa unable to adapt to strong selective pressures, yet we did not observe this result in the microbial communities analyzed here. There could be a number of reasons why we did not observe a shift. First, some bacterial taxa may avoid Se stress. Se is a temporal stress as it enters most of our systems with snowmelt and rain in spring, but we do not observe Se in many of our soils by late spring or summer. Thus, some taxa could form cysts or limit respiration when exposed to Se. Second, some bacterial taxa may retain rarely used Se tolerance genes. For example, a surprising number of bacterial species retain genes for Se respiration despite encountering Se less frequently than other abiotic stresses [46]. Third, bacteria with Se respiration genes could facilitate the survival of neighboring taxa by removing the toxin from the environment. Fourth, selection or other genomic evolution mechanisms act so quickly on bacterial genomes that taxa have adapted before they are eliminated. It is likely that bacterial taxa in these systems utilize one or more of these mechanisms to persist in their toxic environment. Our data were unable to disentangle such mechanisms.

### Changes in gene content

Our second hypothesis stated that we expected to see greater changes in gene content at sites with Se and that genes with recorded changes would be related to Se respiration or tolerance. In support of the first half of this hypothesis, we observed almost three times as many changes in gene content in soils with higher Se. Although only four of these genes (*recG*, *selA*, *selB*, and *serA*) have previously been reported to be related to Se, this is likely due to a lack of gene annotations for processes associated with Se cycling. Se cycling by microbes is a relatively understudied process, thus as the number of studies increases we expect many more genes to be identified. We also observed a number of genes associated with tolerance of other heavy metals. Interestingly, all our soils, regardless of Se content, contain elevated concentrations of other heavy metals, such as magnesium, nickel, manganese, and zinc. We specifically chose matched sites to have soil chemistry that differed primarily in terms of Se (see Supplementary Materials, Tab. S2). Thus, if these genes play a role in promoting tolerance to other heavy metals, we would expect them to be evenly distributed across all our sites. This suggests that these genes also play a role in the microbial tolerance of Se.

The number of genes displaying relative abundance differences between sites is a clear indicator that Se acts as a strong selective pressure on bacteria. Our results are primarily correlative, but the lack of differences in community composition and physiochemical properties between high and low Se sites (Table S2) strongly support our hypothesis that Se is a driving factor in altered gene composition within Se sites. In addition, the large number of genes promoted at high-Se sites indicates that there are multiple pathways involved in Se tolerance within bacteria. To date, only a handful of strategies for tolerating Se have been identified in bacteria, and these strategies primarily involve respiration and oxidation [46]. Yet even within the subset of genes identified here, we identified alternative pathways related to Se tolerance. For example, *RecG* primarily functions as a helicase that acts at junctions [47, 48], allowing for DNA repair, and has been suggested to work with *OxyR* to control the expression of oxidative stress-related genes [49]. *RecG* could, in our system, primarily reduce DNA damage caused by Se-associated compounds [43]. Thus, there could be multiple mechanisms for Se tolerance that do not involve respiration or oxidation or that are not specific to Se.

Although we were not able to record the change in gene content over time, we expect that changes in gene content were driven by the introduction of Se into our sites over only a few months (March–May). This is similar to patterns that have been observed in other soil systems [18, 19], where the evolution of single or multiple species within a soil microbiome has been documented within two to six months. This suggests that evolution within soil systems can be relatively rapid and that evolution in our microbiomes to a single stress (Se) occurred at the same rate as evolution in response to multiple or compound stresses (differences in climates, etc.).

## Conclusions

Our results strongly suggest selection within microbiomes for response to a single strong stressor (Se) via a significant increase in differentially abundant genes within sites with high Se. Unlike in most systems, we observed that the dominant form of adaptation is the accumulation of genes among existing microbial taxa rather than microbial species turnover. This work identifies a unique system in which we can observe evolution in a non-gut natural system and contributes to growing interest in understanding microbiome evolution in non-model systems [6].

## Electronic supplementary material

Below is the link to the electronic supplementary material.

Supplementary Material 1

## Data Availability

The raw data are available at the NCBI SRA under bioprojects PRJNA1080584 (shotgun metagenomics) and PRJNA1080582 (amplicon sequencing).
