# Supplementary material for: Selenium alters the gene content but not the taxonomic composition of the soil microbiome
Source: Environ Microbiome. 2024 Nov 18;19:92. doi: 10.1186/s40793-024-00641-x (PMC11575018; doi:10.1186/s40793-024-00641-x)
Supplement: Supplementary file 1 — Supplementary Material 1 [file 40793_2024_641_MOESM1_ESM.pdf]

Selenium alters the gene content but not the taxonomic composition of the soil microbiome

Alison E. Bennett[1], Scott Kelsey[1], Casey Saup[2], Mike Wilkins[3], Antonino Malacrinò[4,5]

[1] Dept. of Evolution, Ecology, and Organismal Biology, The Ohio State University, Columbus (OH), USA
[2] School of Earth Sciences, The Ohio State University, Columbus (OH), USA
[3] Dept. of Soil and Crop Sciences, Colorado State University, Fort Collins (CO), USA
[4] Dept. of Agriculture, Università degli Studi Mediterranea di Reggio Calabria, Reggio Calabria, Italy
[5] Dept. of Biological Sciences, Clemson University, Clemson (SC), USA
* corresponding author: (AM) antonino.malacrino@unirc.it

Supplementary material

Supplementary tables

Table S1. Samples from paired high/low selenium sampling sites in Western Colorado, USA. For each sample, we provide information about the underlying geological parent material, whether they were classified as High or Low in Selenium, the sampling GPS coordinates, and whether samples were used for shotgun or amplicon sequencing (green-colored cell indicates that the sample was used for that application).

Sample	Formation	Selenium	GPS coordinates	Shotgun	Amplicon
JW2	Wasatch	High	39.32699 N<br>108.26165 W		
JW3	Wasatch	High	39.325620 N<br>108.263270 W		
DMW1	Wasatch	Low	39.32008 N<br>108.16945 W		
DBMH	Wasatch	High	39.31594 N<br>108.17452 W		
MFT1	Mancos	Low	38.51696 N<br>107.85333 W		
SWL1	Mancos	High	38.71129 N<br>108.02534 W		
UNWL	Morrison	Low	38.344400 N<br>108.728850 W		
UVMH	Morrison	High	38.343600 N<br>108.732550 W		
ECW1GL	Morrison	Low	38.66999 N<br>108.33148 W		
GJSRW1H	Morrison	Low	38.99683 N<br>108.61191 W		
GJSRW3	Morrison	High	38.99149 N<br>108.61673 W		
GDWL	Mancos	Low	38.419670 N<br>107.771160 W		
LZSL	Mancos	High	38.539840 N<br>107.788480 W		
LZS3	Mancos	High	38.54470 N<br>107.78506 W		
BLML	Morrison	High	38.45795 N<br>108.84424 W		
BLM4	Morrison	Low	38.45779 N<br>108.84837 W		
EWA1	Mancos	Low	38.70968 N<br>108.17004 W		
TRK3	Wasatch	High	39.30972 N<br>108.27467 W		
TRK4	Wasatch	Low	39.30971 N<br>108.27479 W		
DIGL	Wasatch	Low	39.31537 N<br>108.15984 W		

Supplementary figures

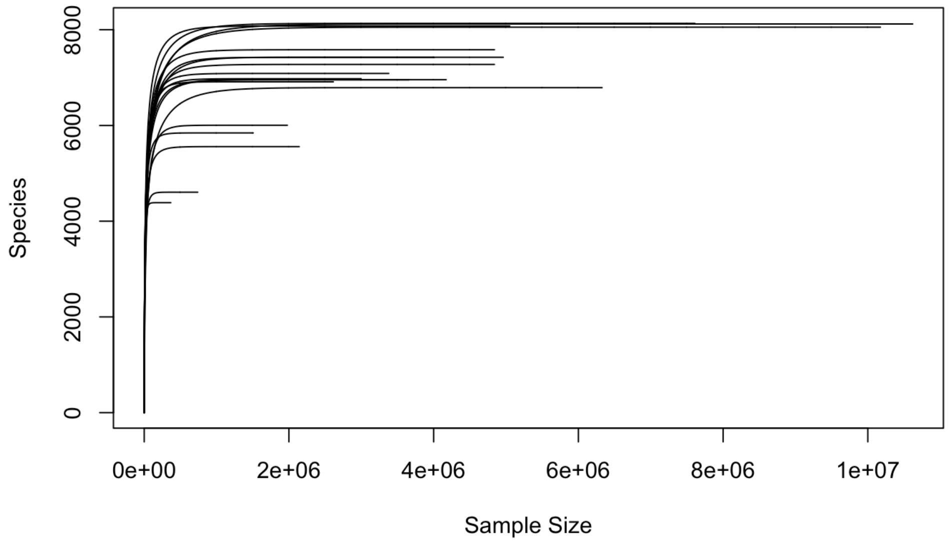

Figure S1. Rarefaction curves from shotgun metagenomics.

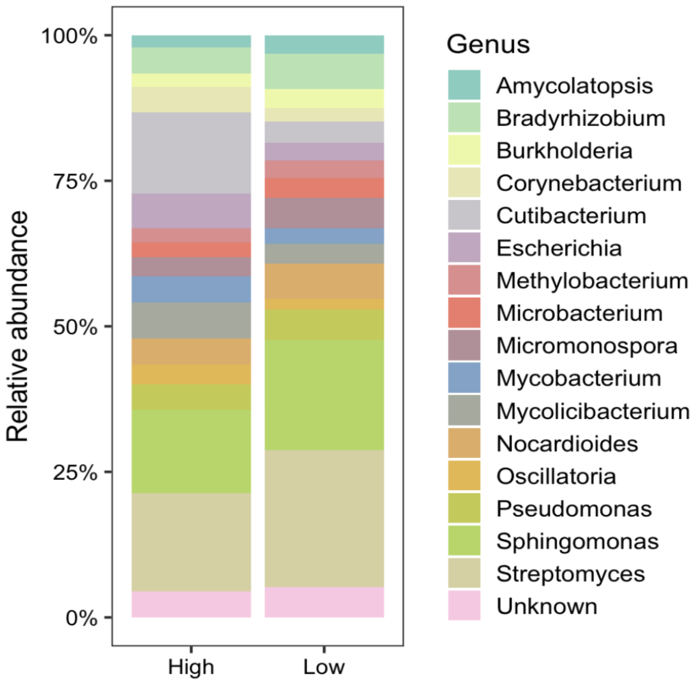

Figure S2. Taxonomic profile of microbial communities in soil with high or low Se from shotgun metagenomics. Microbial genera with relative abundances > 1% are plotted.

Supplementary results – heavy metals in soil

Samples from our sites were submitted to the Kansas State Soil Testing Lab to measure the content of several heavy metals commonly found in soils (Al, As, Cd, Cr, Pb, Cu, Mn, and Zn), total P, total N, and EC. For each, we fit the concentration measure in our sample to a linear mixed-effect model, using the selenium level (high or low) as a fixed factor and geological formation as a random effect.

Table S2. Comparison of concentration of different heavy metals between high and low selenium soils. For each metal, we report the mean concentration (± SE) and the results from the linear mixed-effect model.

**Element**	**High selenium soils (mean ± SE)**	**Low selenium soils (mean ± SE)**	**$\chi 2$**	**p**
Al (ppm)	0.15 ± 0.05	0.23 ± 0.06	0.81	0.37
As (ppm)	3.08 ± 1.09	2.49 ± 0.87	0.31	0.57
Cd (ppm)	1.41 ± 0.57	0.95 ± 0.46	0.57	0.44
Cr (ppm)	7.66 ± 1.75	8.07 ± 2.3	0.03	0.84
Pb (ppm)	10.50 ± 1.18	8.83 ± 0.16	1.44	0.22
Cu (ppm)	0.83 ± 0.13	0.47 ± 0.11	6.12	0.01
Mn (ppm)	2.98 ± 0.59	3.41 ± 0.49	0.30	0.58
Zn (ppm)	1.49 ± 0.57	0.40 ± 0.11	3.43	0.06
Total P (%)	530 ±142	323 ± 66.2	2.20	0.13
Total N (%)	0.14 ± 0.04	0.06 ± 0.01	2.76	0.09
EC (%)	20.07 ±11.4	1.07 ± 0.42	4.11	0.04

Supplementary results – amplicon sequencing

Sampling

The sampling protocols were the same as those reported in the methods in the main document; however, fewer total sites were sampled.

DNA extraction, library preparation, and sequencing

Total DNA was extracted from soil samples using the Powersoil DNA Isolation Kit (MoBio Laboratories, CA, USA). Bacterial communities were characterized through 16S rRNA gene sequencing using primers 515f/806r that target the V4 region and maximize coverage of bacteria and archaea while also providing polymerase chain reaction products long enough for sequencing (Apprill *et al.*, 2015; Parada *et al.*, 2016). These 16S rRNA genes were sequenced using an Illumina MiSeq instrument at Argonne National Laboratory.

Data processing and analysis

The data were processed using the nf-core/ampliseq version 2.7.1 (doi: 10.5281/zenodo.1493841). Cutadapt (Martin, 2011) was used to trim primer sequences, which were then processed sample-wise with DADA2 (Callahan *et al.*, 2016) to eliminate PhiX contamination, discard reads with > 2 expected errors, correct errors, merge read pairs, remove chimeras, and identify amplicon sequence variants (ASVs).

Data were then processed using R v4.1.2 (R Core Team, 2020) and phyloseq v1.38 (McMurdie and Holmes, 2013). All singletons were then discarded. The Shannon diversity index was calculated using the package microbiome v1.16 (Sudarshan and Shetty, 2017), and differences between high- and low-selenium soils were tested by fitting a linear mixed-effects model with the package lme4 (Bates *et al.*, 2014) using the selenium level (high or low) as a fixed factor and geological formation as a random effect. We also tested the effect of the selenium level (high or low) on the structure of microbial communities using PERMANOVA on a Bray–Curtis distance matrix between samples (999 permutations, stratified using the variable "geological formation"). Differences in the structure of the soil microbial communities were visualized using NMDS. Data were then normalized using Wrench (Kumar *et al.*, 2018), and using the package MaAsLin2 (Mallick *et al.*, 2021), we identified taxa with differential abundance between soils with high and low Se.

Results

Our results showed that the presence of high levels of Se did not influence the diversity (χ^2=2.08, p=0.14; Fig. S3A) or structure ($F_{1,8}$=1.08, p=0.23; Fig. S3B) of the soil microbial communities. Similarly, when testing for differences in individual ASVs between the two soil groups, we did not find any taxa that were significantly more abundant in soils with high or low levels of Se (Fig. S3C).

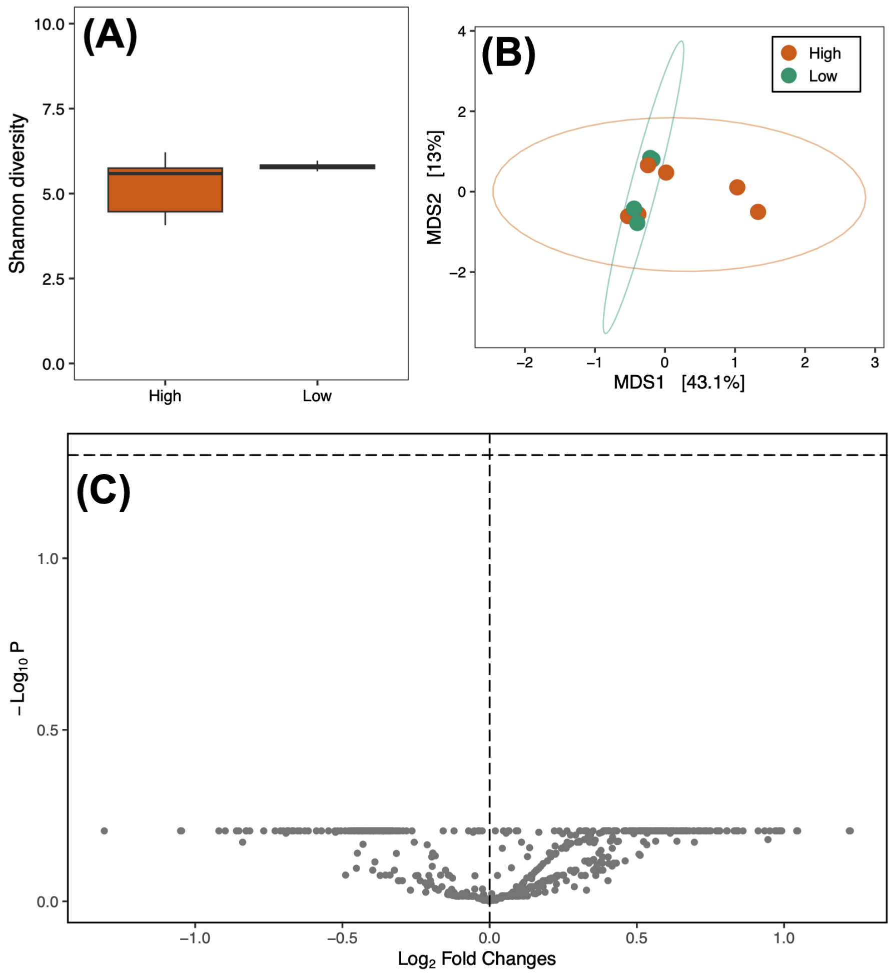

Figure S3. (A) Shannon diversity index in soil samples with high (n = 6) and low (n = 4) Se. (B). NMDS (Non-Metric Multi Dimensional Scaling) of soil samples with high (n = 6) and low (n = 4) selenium levels. The ellipses represent the 95% CIs for each group. (C) Volcano plots showing that no microbial taxa were differentially abundant between sites with high (log2FC > 0) and low (log2FC < 0) Se levels.

References

Apprill, A., McNally, S., Parsons, R., and Weber, L. (2015) Minor revision to V4 region SSU rRNA 806R gene primer greatly increases detection of SAR11 bacterioplankton. *Aquat Microb Ecol* **75**: 129–137.

Bates, D., Mächler, M., Bolker, B., and Walker, S. (2015) Fitting linear mixed-effects models using lme4. *Journal of statistical software* **67**: 1–48.

Callahan, B.J., McMurdie, P.J., Rosen, M.J., Han, A.W., Johnson, A.J.A., and Holmes, S.P. (2016) DADA2: High-resolution sample inference from Illumina amplicon data. *Nat Methods* **13**: 581–583.

Kumar, M.S., Slud, E.V., Okrah, K., Hicks, S.C., Hannenhalli, S., and Corrada Bravo, H. (2018) Analysis and correction of compositional bias in sparse sequencing count data. *BMC Genomics* **19**: 799.

Mallick, H., Rahnavard, A., McIver, L.J., Ma, S., Zhang, Y., Nguyen, L.H., et al. (2021) Multivariable association discovery in population-scale meta-omics studies. *PLoS Comput Biol* **17**: e1009442.

Martin, M. (2011) Cutadapt removes adapter sequences from high-throughput sequencing reads. *EMBnet J* **17**: 10.
McMurdie, P.J. and Holmes, S. (2013) phyloseq: an R package for reproducible interactive analysis and graphics of microbiome census data. *PLoS One* **8**: e61217.
Parada, A.E., Needham, D.M., and Fuhrman, J.A. (2016) Every base matters: assessing small subunit rRNA primers for marine microbiomes with mock communities, time series and global field samples. *Environ Microbiol* **18**: 1403–1414.
R Core Team (2020) R: A language and environment for statistical computing.
Sudarshan, L. and Shetty, L. (2017) Tools for microbiome analysis in R.
